# Piezo-tolerant natural gas-producing microbes under accumulating *p*CO_2_

**DOI:** 10.1186/s13068-016-0634-7

**Published:** 2016-11-04

**Authors:** Ralph E. F. Lindeboom, Seung Gu Shin, Jan Weijma, Jules B. van Lier, Caroline M. Plugge

**Affiliations:** 1 Sub-Department of Environmental Technology, Wageningen University, P.O. Box 8129, 6700 EV Wageningen, The Netherlands; 2 Section Sanitary Engineering, Department of Water Management, Faculty of Civil Engineering and Geosciences, Delft University of Technology, P.O. Box 5048, 2600 GA Delft, The Netherlands; 3 School of Environmental Science and Engineering, Pohang University of Science and Technology, 77 Cheongam-ro, Nam-gu, Pohang, Gyeongbuk 37673 South Korea; 4 Laboratory of Microbiology, Wageningen University, Stippeneng 4, 6708 WE Wageningen, The Netherlands

**Keywords:** Autogenerative high-pressure digestion, Population dynamics, Syntrophy, Propionate accumulation, CO_2_-toxicity, Gibbs free energy, Carboxylate platform

## Abstract

**Background:**

It is known that a part of natural gas is produced by biogenic degradation of organic matter, but the microbial pathways resulting in the formation of pressurized gas fields remain unknown. Autogeneration of biogas pressure of up to 20 bar has been shown to improve the quality of biogas to the level of biogenic natural gas as the fraction of CO_2_ decreased. Still, the *p*CO_2_ is higher compared to atmospheric digestion and this may affect the process in several ways. In this work, we investigated the effect of elevated *p*CO_2_ of up to 0.5 MPa on Gibbs free energy, microbial community composition and substrate utilization kinetics in autogenerative high-pressure digestion.

**Results:**

In this study, biogas pressure (up to 2.0 MPa) was batch-wise autogenerated for 268 days at 303 K in an 8-L bioreactor, resulting in a population dominated by archaeal *Methanosaeta concilii*, *Methanobacterium formicicum* and *Mtb. beijingense* and bacterial *Kosmotoga*-like (31% of total bacterial species), *Propioniferax*-like (25%) and *Treponema*-like (12%) species. Related microorganisms have also been detected in gas, oil and abandoned coal-bed reservoirs, where elevated pressure prevails. After 107 days autogeneration of biogas pressure up to 0.50 MPa of *p*CO_2_, propionate accumulated whilst CH_4_ formation declined. Alongside the *Propioniferax*-like organism, a putative propionate producer, increased in relative abundance in the period of propionate accumulation. Complementary experiments showed that specific propionate conversion rates decreased linearly from 30.3 mg g^−1^ VS_added_ day^−1^ by more than 90% to 2.2 mg g^−1^ VS_added_ day^−1^ after elevating *p*CO_2_ from 0.10 to 0.50 MPa. Neither thermodynamic limitations, especially due to elevated pH_2_, nor pH inhibition could sufficiently explain this phenomenon. The reduced propionate conversion could therefore be attributed to reversible CO_2_-toxicity.

**Conclusions:**

The results of this study suggest a generic role of the detected bacterial and archaeal species in biogenic methane formation at elevated pressure. The propionate conversion rate and subsequent methane production rate were inhibited by up to 90% by the accumulating *p*CO_2_ up to 0.5 MPa in the pressure reactor, which opens opportunities for steering carboxylate production using reversible CO_2_-toxicity in mixed-culture microbial electrosynthesis and fermentation.

**Graphical abstract:**
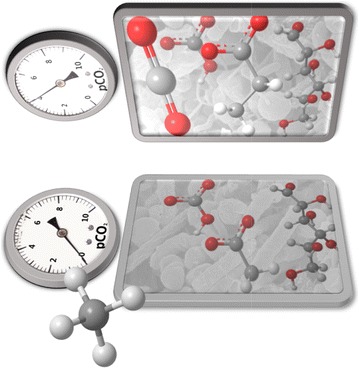
The role of *p*CO_2_ in steering product formation in autogenerative high pressure digestion

**Electronic supplementary material:**

The online version of this article (doi:10.1186/s13068-016-0634-7) contains supplementary material, which is available to authorized users.

## Background

Natural gas is a non-renewable fossil fuel formed over thousands of years in a distant past. Currently, shale gas, coal-bed gas, biogas and clathrates are highlighted to replace the declining resources from ancient natural gas fields [1–4]. Isotope measurements have confirmed that natural gas was partially produced by either thermogenic cracking or biogenic degradation of organic matter [5, 6], but the microbial pathways resulting in the formation of pressurized gas fields have not been explored. Biogas from anaerobic digesters consists of the same key components CH_4_, CO_2_, H_2_S and H_2_O as natural gas and is produced from organic matter by mixed-culture microbial fermentation. Anaerobic microorganisms that originate from non-pressurized digesters can autogenerate biogas pressure of up to 9.0 MPa [7] and convert maize silage in a two-phase pressurized digester [8]. There is an indication that even higher pressures can be autogenerated, but pressure-sensitive equipment has limited our ability to investigate the upper limits of pressure. This raises the question of a relation between microbial communities enriched in high-pressure anaerobic digesters today and those involved in the formation of ancient biogenic natural gas fields such as the Groningen gas reservoir in the Netherlands, which had an initial pressure of 35 MPa. Multiple researchers have isolated methanogenic archaea, such as *Methanobacterium (Mtb) formicicum* and *Methanosaeta (Mst.)* (*Methanothrix*) *concilii,* from high-pressure subsurface gas and oil reservoirs [9, 10]. From this perspective, understanding the microbial pathways and population dynamics in autogenerative high-pressure digestion (AHPD) is fascinating and relevant not only from a technological point of view, but also by offering potential insight into the origin of biogenic natural gas and the consequences of carbon capture in subsurface reservoirs [11].

At elevated biogas pressure, more CO_2_ and H_2_S remain dissolved in the water phase due to Henry’s law. Other than the accumulation of the notorious inhibitor H_2_S, the accumulation of CO_2_ in water is also critical because of the inhibitory effect on microorganisms at elevated concentrations, a fact often utilized in known for example from food preservation [12, 13]. CO_2_ delays growth of pathogens and interferes with the metabolic pathways [11, 12, 14]. CO_2_ can not only serve as the electron acceptor in microbial metabolism (both anabolism and catabolism), but is also an intermediate or an end-product in fermentations. However, as far as we know, the effect of *p*CO_2_ at elevated pressures on individual anaerobic microorganisms has not been quantified; a 30% inhibition on anaerobic digestion of sodium acetate was found under a *p*CO_2_ of 0.10 MPa [15] and 9.00 MPa biogas pressure [7].

**Fig. 1:**
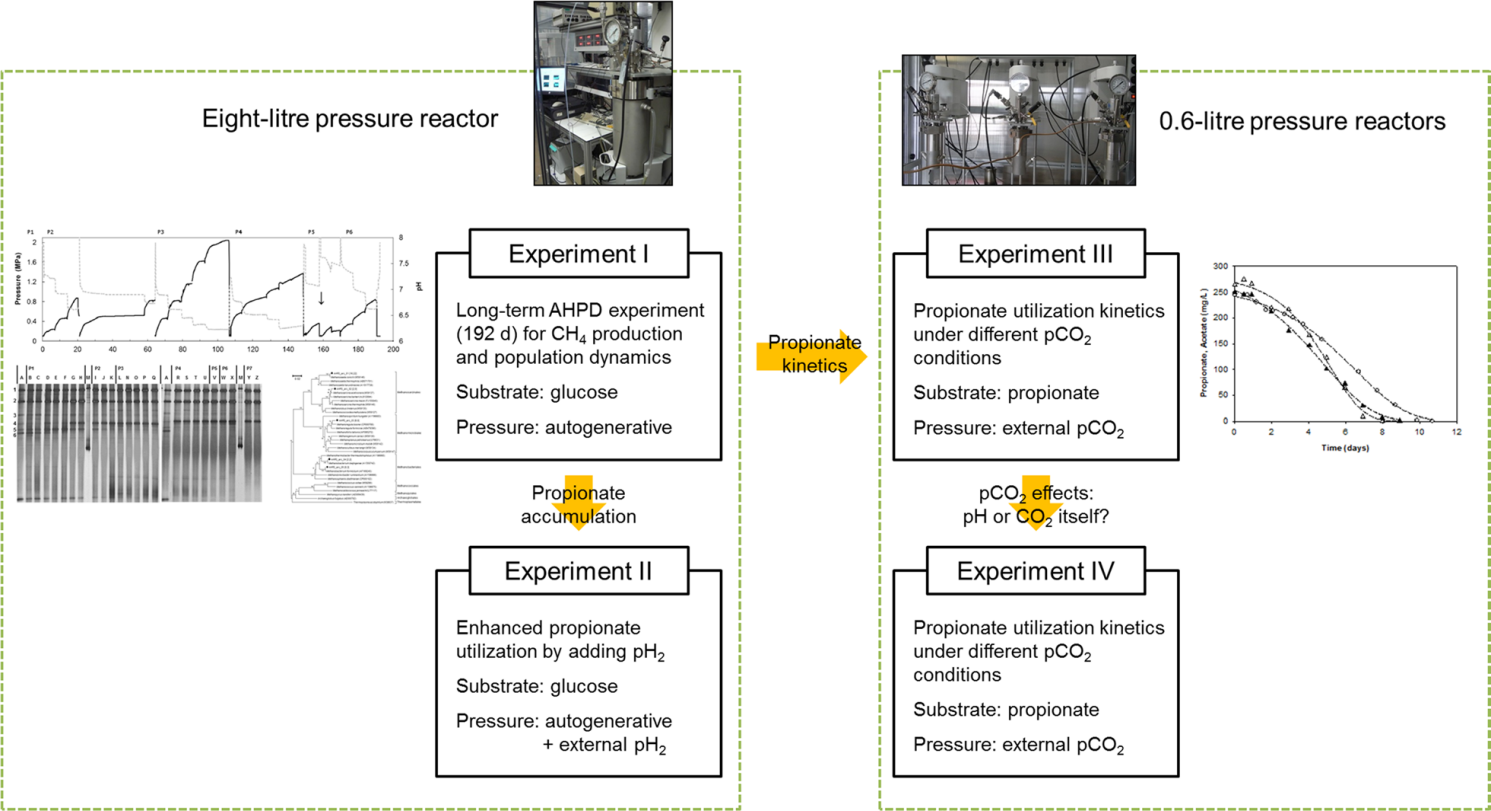
Overview of experimental design

In this study we explore the effect of AHPD conditions, especially *p*CO_2_ on population dynamics and the conversion of glucose. The experimental plan was divided into four sequential experiments. Experiment 1 was explorative and studied CH_4_ production and population dynamics in an 8-L bioreactor in which glucose was digested to 2 MPa biogas over 192 days. As the anaerobic conversion of propionate proved to represent the most critical step, experiment 2 focused on enhancing propionate utilization in the 8-L reactor using added pH_2_ in light of thermodynamic expectations. Experiment 3 then aimed to test our hypothesis on a relation between observed propionate conversion kinetics and different *p*CO_2_ conditions in 0.6-L reactors using the pressure cultivated sludge. Experiment 4 was designed to clarify to which extent the observed propionate conversion kinetics could be attributed to a pH or *p*CO_2_ mechanism (Fig. 1).

## Methods

### Experimental setup of 8- and 0.6-L reactors

An 8-L AHPD reactor setup (Parr Instruments, model 910908, The Netherlands) as described elsewhere [16] was used for this study. All experiments were performed at 30 °C using a water bath (Julabo MP). Total pressure (Ashcroft A-series 1000 PSI), temperature (PT100) and pH (high-pressure pH probes, Bűchi Labortechnick AG, Flawil, Switzerland) were measured online and data were logged with Compact field point modules (cFP1804 and cFP-CB1) and stored with Labview 7.1 PC-software (National Instruments Corporation, USA). The 0.6-L reactor contained Prosense high-pressure pH probes (Prosense serial nr. 34002/002). Two six-bladed impellers attached to a central stirrer shaft (type A709HC, Parr Instruments, The Netherlands) were used to stir the reactors continuously at 150 rpm for the 8-L reactor and 60 rpm for the 0.6-L reactors.

### Experiment I: pressure cultivation of the microorganisms

The mesophilic anaerobic conversion of glucose was studied in the 8-L bioreactor operated at a liquid volume of 6.5 L and a gas volume of 1.5 L over the course of 1 year. The reactor was controlled at a constant temperature (303 K, 30 °C). The overall cultivation was divided into six separate periods: (P1) adaptation to a sodium concentration of 3.5 g Na^+^ L^−1^, (P2) adaptation to autogenerated pressure conditions on glucose, (P3) pressure operation A on glucose, (P4) pressure operation B on glucose, (P5) reactor recovery and (P6) pressure operation C on glucose (Table 1).

**Table 1:** Overview of AHPD-experiments

Exp nr.	Experiment	Headspace	Period	P_start_	Sludge	Substrate		DNA sample
	Description	composition	Days^**a**^	MPa	g VS L^-1^	Type	g COD reactor^-1^	
0-0	Inoculum							A
0-1 Till	P1) Sodium adaptation	Autogenerated	−110	0.10	2.0 –	NaAc –	6.4 –	B till
0-10			−14	0.10	2.9	NaAc	6.4	H
I-1	P2) Glucose and pressure adaptation	Autogenerated	0–7	0.10	2.9	Glucose	7.2	I
I-2			7–14	0.27		Glucose	7.2	
I-3			14–21	0.60	4.0	Glucose	7.2	J
I-4			21–56	0.10		Glucose	14.4	
I-5			56–63	0.65	3.8	Glucose	7.2	K
I-6	P3) High pressure operation- A	Autogenerated	63–70	0.10	4.0	Glucose	14.4	L
I-7			70–77	0.60	4.7	Glucose	14.4	N
I-8			77–84	0.90	5.5	Glucose	14.4	O
I-9			84–93	1.22	6.3	Glucose	14.5	P
I-10			93–107	1.68	7.1	Glucose	14.4	Q
I-11	P4) High pressure operation- B	Autogenerated	107–114	0.10	2.0	Glucose	14.4	R
I-12			114–128	0.62	4.0	Glucose	14.4	S
I-13			128–135	0.88	5.0	Glucose	7.2	T
I-14^b^			135–149	1.06	3.6	gluc + HAc	14.4	U
I-15^b^	P5) Reactor Recovery	Autogenerated	149–157	0.10		gluc + HAc	7.2	V
I-16^b^		pH_2_	157–169	0.10		HAc + H_2_	3.6 + 0.1^c^	
I-17	P6) High pressure operation- C	Autogenerated	169–176	0.10		Glucose	7.2	
I-18			176–183	0.35		Glucose	7.2	W
I-19			183–192	0.64		Glucose	14.4	X
II-1	Stimulation Propionate degradation in eight-litre reactor	Autogenerated	248–257	0.10		Glucose	14.4	
II-2		pH_2_	257–261	0.30		H_2_	0.27^c^	
II-3		pH_2_	261–268	0.36		H_2_	0.40^c^	
III-1	Kinetics Propionate degradation in 0.6-litre reactors	pCO_2_	268–290	0.00	2.2	Propionate	0.37	
III-2				0.10	2.2	Propionate	0.37	Y
III-3				0.30	2.2	Propionate	0.37	
III-4				0.50	2.2	Propionate	0.37	
IV-1	pH-effect Propionate degradation pH 8.0	pN_2_	290–297	0.10	1.0	Propionate	1.8	
IV-2		pCO_2_		0.05	1.0	Propionate	1.8	
IV-3	pH-effect Propionate degradation pH 6.3	pN_2_		0.10	1.0	Propionate	1.8	Z
IV-4		pCO_2_		<0.60	1.0	Propionate	1.8	

^a^medium addition and total sampling liquid were equal to keep 1.5 L gas phase

^b^HAc = undissociated acetic acid was added to keep ANC constant, but directly dissociated due to excess HCO_3_^−^

^c^concerns manual addition of pH_2_ (MPa)

Mesophilic anaerobic granular sludge from an expanded granular sludge bed (EGSB) reactor processing fruit juice wastewater (Friesland Campina, Ede, The Netherlands) was used as inoculum (2 g VS L^−1^). Liquid medium with yeast extract, trace elements and macro-nutrient solution was provided as previously described [7].

In previous experiments [17], we found that methanogenic activity on acetate of the inoculum sludge was sensitive to sodium in the range of 0.9–3.6 g Na^+^ L^−1^. Therefore, sodium acetate was fed in period 1 (P1) to allow adaptation of the acetotrophic population to sodium under atmospheric conditions (P1 experiment 0–1 to 0–10, Table 1). Addition of sodium acetate resulted in the acid-neutralizing capacity (ANC) of 150 meq NaHCO_3_ L^−1^, which was maintained constant throughout the further experiments in this reactor.

From period 2 (P2 experiment I-1 to I-5, Table 1) to period 6 (P6 experiment I-17 to I-19), glucose was fed as substrate. Fresh substrate was fed in a concentrated 50 mL solution to compensate for all sampling losses and keep the liquid volume constant. d-Glucose (Merck) was dissolved in 50 mL of fresh liquid medium. 7.5 mmol of NaHCO_3_ was added to maintain the ANC at 150 meq L^−1^ to compensate for the sampling losses and keep the carbonate equilibrium stable. Gas samples were taken perpendicular to the gas flow direction whilst pressure was released using a needle valve, as described in detail in previous work [16]. Liquid samples for TS/VS, VFA and microscope analysis were taken using a dip tube from the centre of the pressurized reactor vessel, whilst stirring. The initial 5 mL of sample were discarded (the dead volume of the diptube) to ensure that it represented the bulk composition. VFA and biogas samples were always taken in duplicate and the frequency was adjusted (varying from once per few hours to once a 1-week interval) according to the observed pressure dynamics. With this we minimized pressure losses, as each sampling moment caused a pressure decrease (0.01–0.03 MPa loss per sample).

### Experiments II, III and IV: propionate degradation in 8- and 0.6-L reactors

Experiment II focused on propionate accumulation and conversion under elevated autogenerated biogas pressure. In experiment II-1 propionate accumulated, and in II-2 and II-3 hydrogen was added as the substrate to stimulate the hydrogenotrophic population and facilitate subsequent propionate oxidation due to enhanced H_2_-scavenging.

Propionate conversion under different *p*CO_2_ (0.0, 0.1, 0.3, and 0.5 MPa) was then studied using batch cultures (experiment III) at a temperature of 303 ± 1 K or 30 °C. (Table 1). The batch incubation at elevated *p*CO_2_ (0.3 and 0.5 MPa) was done in 0.6-L steel bioreactors with 0.2 L liquid volume [18] and the atmospheric (unpressurized) incubation in 0.125-L glass serum bottles with 0.05 L liquid volume. The seed sludge, 10.8 ± 0.3 g VS L^−1^, was taken from the 8-L reactor at the end of experiment II-3. The synthetic medium consisted of macronutrients, trace elements [7] and propionate (377.5 mg COD L^−1^ (250 mg L^−1^) at *t* = 0), and the pH was adjusted to 7.0 with 15% HCl. The incubation was started by mixing 20% (v/v) seed sludge and 80% (v/v) medium and replacing the headspace with either 0.1 ± 0.01 MPa (*p*N_2_), 0.10 ± 0.01, 0.30 ± 0.01, or 0.50 ± 0.02 MPa *p*CO_2_. Additional CO_2_ was injected in the period of initial CO_2_ dissolution to maintain the *p*CO_2_ at the desired level. Liquid samples were taken from the cultures to quantify volatile fatty acids (VFAs). When propionate was below the detection limit, gas composition was analysed with a gas chromatograph (GC) to calculate conversion efficiency. Lag periods and propionate degradation rates were calculated using the modified Gompertz model (Eq. 1) [19].

1 $$ y = A \exp \left\{ { - \exp \left[ {\frac{{r_{\text{smax} } \cdot \exp (1)}}{A}\left( {\lambda - t} \right) + 1} \right]} \right\} $$

where *A* is the maximum value of propionate concentration (near to the initial value), *r*_smax_ maximum substrate utilization rate (mg COD L^−1^ day^−1^), and *λ* lag time.

Additional experiments (experiment IV) with 1 g VS L^−1^ pressure cultivated inoculum (from experiment II-3), and 1.8 g propionate L^−1^ were performed in duplicate to determine whether inhibition effects could be explained by decreasing pH or elevated *p*CO_2_ related (Table 1).

### Analytical procedures

A limited number of biogas samples were taken from the head space to minimize biogas losses, with samples taken under stable pressure. Liquid was collected in a closed sampling bottle for determining dissolved CH_4_, CO_2_(aq) and HCO_3_^−^ concentrations after [16]. Biogas samples were injected into a GC (Shimadzu GC-2010, Kyoto, Japan) at atmospheric pressure using 0.4 MPa He as the carrier gas whilst directing the sample over two columns: a Molsieve (Alltech 13940) and Porabond Q (Varian 7354) for CH_4_, CO_2_, N_2_ [7]. H_2_ was measured with in an HP5980A gas chromatograph (Hewlett Packard, Palo Alto, alto, USA) and directed over a molsieve column using argon gas as carrier [17]. Both GCs used a thermal conductivity detector. Biogas samples were taken from the gas phase and by gas expansion from the liquid phase. Biogas composition was corrected for flush gas (N_2_) and water vapour (data from standard tables) and showed a deviation from the mean of less than 2% (duplicate samples). After biogas measurements from the expansion sample vessel, sampling bottles were opened and the acid-neutralizing capacity (ANC) was determined by end-point titration (pH 4.1). HCO_3_^−^ values were corrected for measured VFA.

VFAs were measured by gas chromatography (Hewlett Packard 5890 series II, Palo Alto, USA) using a glass column coated with Fluorad 431 on a Supelco-port (mesh 100–120) with a flame ionization detector as described previously [7].

A high-performance liquid chromatograph (HPLC; Dionex Corporation, Sunnyvale, USA) was used to determine the concentration of various dissolved organic intermediates (i.e. fatty acids, organic acids and alcohols) as described elsewhere [20]. Liquid samples were centrifuged at 10,000 rcf and the supernatant of the sample was diluted 1.1–4 times, depending on expected VFA concentrations to a H_2_SO_4_ vial concentration of 0.2 M H_2_SO_4_, a value warranting undissociated VFAs by the elimination of the buffering capacity of 150 mM HCO_3_^−^. Samples were eluted via an autosampler with 1.25 mM H_2_SO_4_, injected and pumped at a flow rate of 0.6 mL min^−1^ with an HPLC pump (Dionex High Precision model 480) separated on an Alltech OA-1000 column (length = 300 mm, internal diameter = 6.5 mm) at 60 °C and 6.0–6.5 MPa and detected by means of refractive index.

Total solids (TS) and volatile solids (VS) were determined after [21] instead of total suspended solids (TSS) and volatile suspended solids (VSS), because visually suspended biomass (after centrifugation) showed to pass through the filters.

Samples for field emission scanning electron microscopy (FeSEM) were centrifuged for 10 min at 4300 rcf. Hereafter, supernatant was replaced by a 2.5% (w/v) glutaraldehyde solution for fixation for 1 h at 4 °C. Samples were then dehydrated in a series of ethanol 50–75–90–95–100% and transferred to acetone. To prevent the samples from shrinking due to removing the acetone in air, a supercritical carbon freeze drying procedure was used [22]. The samples were then glued to a brass sample holder with iridium glue. Then samples were sputter-coated with iridium. The field emission scanning electron microscope (Fei Magellan FESEM) was connected to an Oxford Aztec EDX and operated between 2 kV and 6.3 pA current. Scattered electrons were detected by Through Lens Detection (TLD) at a working distance of 1.9 and 5.1 mm.

### DNA extraction and amplification

Samples were centrifuged at 10,000 rcf for 5 min and stored in RNAlater (Life Technologies, Carlsbad, CA) at −20 °C before DNA extraction. Total genomic DNA was extracted using FastDNA Spin kit for soil (MP Biomedicals, Santa Ana, CA). The extracted DNA was quantified and checked for purity with a Nanodrop spectrophotometer (Nanodrop Technologies, Wilmington, DE). The 16S rRNA genes were amplified using Phire Hot Start DNA polymerase (Thermo Fisher Scientific, Vantaa, Finland). For DGGE, primer pairs GC-ARC344f/519r [23] and GC-968f/1401r [24] were used to amplify the archaeal and bacterial 16S rRNA genes, respectively. The PCR mixture of 50 μL contained 400 nM of each primer, 200 μM of dNTP and 50 ng of template DNA. PCR was performed according to the following thermocycling protocol: pre-denaturation at 98 °C for 2 min; 35 cycles of denaturation at 98 °C for 10 s, annealing at 56 °C for 10 s, and elongation at 72 °C for 20 s (Archaea) or 30 s (bacteria); post-elongation at 72 °C for 10 min. PCR product size was confirmed by electrophoresis in 1% (w/v) agarose gels stained with SYBR Safe (Invitrogen, Carlsbad, CA, USA).

For cloning, nearly full-length 16S rRNA gene fragments amplified with primers 109f and 1492r (Archaea) or 27f and 1492r (Bacteria) were obtained using PCR. The PCR mixture of 50 μL was prepared using the same composition as above, except that GoTaq (Promega, Madison, WI) was used instead of Phire Hot Start DNA polymerase. The thermocycling protocol consisted of pre-denaturation at 95 °C for 2 min; 30 cycles of denaturation at 95 °C for 30 s, annealing at 52 °C for 40 s, and elongation at 72 °C for 90 s; post-elongation at 72 °C for 3 min.

### DGGE

DGGE analysis of the amplicons was conducted on 8% (w/v) polyacrylamide gels with denaturant gradients of 40–60% and 30–60% for archaeal and bacterial communities, respectively, where 100% was defined as 7 M urea with 40% (v/v) formamide. Electrophoresis was performed using a D-Code system (Bio-Rad, Hercules, CA) in 0.5× TAE buffer at 60 °C and 85 V for 16 h. During the first 10 min of the electrophoresis, a voltage of 200 V was applied. The band patterns of the resulting gels were visualized by silver staining [25]. The band intensity was calculated with LabWorks program (version 4.0.0.8; UVP BioImaging Systems) and the heat map of relative band intensity was generated using program package R.

### Clone library and phylogenetic analysis

Clone libraries of 16S rRNA genes were constructed to identify dominant microbial species. Two (A and L, Table 1) and three (F, L, and U, Table 1) DNA samples were chosen for archaeal and bacterial analyses, respectively, to maximize likelihood of including clones related to prominent DGGE bands. Nearly full-length 16S rRNA gene fragments were cloned into pGEM-T easy vector (Promega, Madison, WI) and transformed into *Escherichia coli* DH5α. White colonies were sent for sequencing with the primers SP6 and T7 to GATC Biotech (Konstanz, Germany). All overlapping reads were trimmed of the vector sequences and bad-quality sequences and were assembled into contiguous reads using DNAMAN software (Lynnon Biosoft, Quebec, Canada). Possible chimeras were removed using the Greengenes Bellerophon Chimera check [26]. All sequences were grouped into operational taxonomic units (OTUs) within >97% similarity by constructing a similarity matrix with ClustalX 2.1 [27]. Phylogenetic trees were constructed using neighbour-joining method using MEGA software [28]. Hierarchical classification of the 16S rRNA gene sequences was assisted by classifier from the Ribosomal Database Project [29]. The nucleotide sequences reported in this study have been deposited under GenBank accession numbers KJ206630–KJ206896. Additional DGGE analyses were conducted to crosslink band patterns with identified clones. At least one clone from each OTU was used as a template for amplification using above-mentioned method, using DGGE primer sets. For bacterial clones, a nested PCR approach with SP6 and T7 primers was employed to exclude the amplification of the host 16S rRNA gene. The migration of clonal amplicons was directly compared to that of different bands on denaturing gradient gels.

### Calculations

Based on measured CO_2_ and CH_4_ speciation, the total inorganic carbon (TIC) and methane (TCH_4_) balances were constructed after [16] using the equations described below. Deviation between the measured biogas CO_2_-speciation and theoretical composition based on the measured ANC values were an indication of accumulating fatty acids.

$$ {\text{TIC}} = {\text{HCO}}_{3}^{{ - }} + {\text{ CO}}_{2} ({\text{diss}}) \, + {\text{ CO}}_{2} ({\text{gas}}) $$

or

$$ {\text{TIC}} = ({\text{ANC}}) + \frac{{({\text{ANC}})* 10^{{ - {\text{pH}}}} }}{{K_{1} }} + \frac{{({\text{ANC}})*10^{{ - {\text{pH}}}}  V_{\text{g}} }}{{K_{1} K_{{{\text{HCO}}_{2} }} V_{\text{l}} *R*T}} $$

In which, ANC is given in meq L^−1^, $K_1=10^{-{\rm p}{K_a}}$, $K_{{{\text{HCO}}_{2} }} = 10^{ - 6.55}$  mol L^−1^ Pa^−1^, *V*_l_ = liquid volume in L, *V*_g_ = gas volume in L, *T* = 303 K and *R* = 8.3145 × 10^3^ L Pa K^−1^ mol^−1^.

$$ {\text{TCH}}_{4} = {p\text{CH}}_{4} *\left( {\frac{{V_{\text{g}} }}{R*T} + K_{{HCH_{4}  }} *V_{\text{l}} } \right), $$

where $K_{{\rm HCH}_4}$ = 10^−7.84^ mol L^−1^ Pa^−1^

Based on the TCH_4_ for each time point, volumetric CH_4_ production rates were determined by the differences between time *t*_0_ and *t*_*n*_.

The COD was not measured in this study, but the theoretical COD values (according to the Buswell equation) for CH_4_ (64 g COD mol^−1^ CH_4_), acetate (1.07 g COD g^−1^ acetate), and propionate (1.51 g COD g^−1^ propionate) were used for calculations.

Both *p*CO_2_ and HCO_3_^−^ are commonly used for Gibbs free energy calculations [30, 31]. Because of the changes in CO_2_ speciation due to reactor operation, $\Delta {\text{G}}_{\text{r}}^{{\prime \prime }}$ values for CO_2_(g), CO_2_(aq) and HCO_3_^−^ were calculated for each relevant reaction according to Thauer et al. [32]. Correction for temperature and actual reactor concentrations was performed using data [33] (Additional file 1: Table S1) on the enthalpy of formation (Δ*H*_f_^o^) and the free energy of formation (Δ*G*_f_^o^).

## Results

### Overall reactor performance

**Fig. 2:**
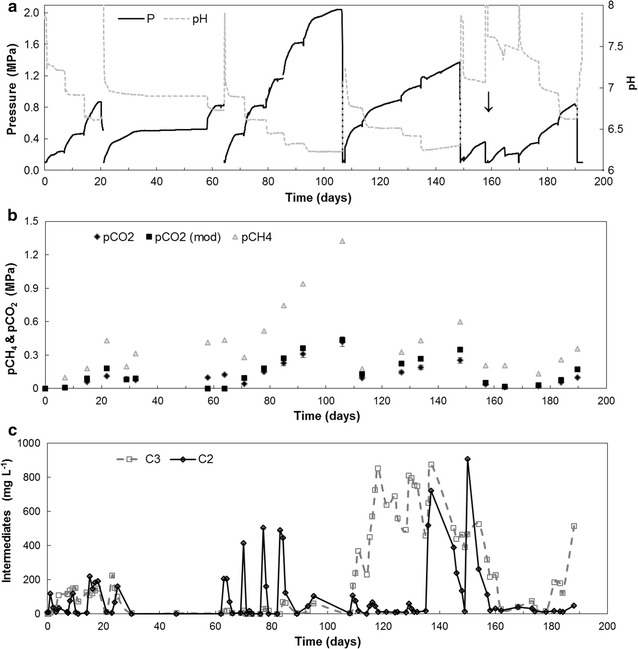
Results of fed-batch reactor operation. **a** Pressure and pH, **b** measured *p*CH_4_, measured *p*CO_2_ and calculated *p*CO_2_, **c** acetate and propionate; *downward arrow* indicates H_2_ addition; P1–P6 indicate operational periods as described in Table 1

Biogas was produced during the first 107 days (until the end of period 3) according to stoichiometry and autogenerated pressures reached 2.0 MPa (Fig. 2a, raw data in Additional file 2). The biogas had an improved CH_4_-content of 75–86% and the maximum volumetric CH_4_-production rate was ~11 mmol L^−1^ day^−1^ or 700 mg COD L^−1^ day^−1^ in period 3 (P3). *p*CO_2_ production contributed for 14–25% to the autogenerated pressure (Fig. 2b). The pH-(**1a**) and the *p*CO_2_-profile (**1b**) mirrored each other and the close proximity of the calculated *p*CO_2_ and measured *p*CO_2_ values indicates that *p*CO_2_ and not VFA was determining the pH.

Acetate and propionate were only formed transiently during the first 100 days and were fully converted into biogas (Fig. 2c). A deviation started to occur between calculated *p*CO_2_ and measured *p*CO_2_ when the propionate started to accumulate after 105–110 days. Propionate has a lower p*K*_a_ than HCO_3_^−^ and forces CO_2_ out of its ionized form into the gas phase and results in a reduced biogas quality [16].

After reaching 1.80–2.00 MPa with a *p*CO_2_ of 0.44 MPa and a pH 6.2 (Fig. 2b), volumetric CH_4_ production decreased to 6–8 mmol L^−1^ day^−1^ or 400–500 mg COD L^−1^ day^−1^, whilst propionate remained below 100 mg L^−1^. Nevertheless, feeding of experiment I-11 was postponed till day 107, due to this decrease.

Pressure autogeneration was repeated in period 4 (P4 day 107–135) after decreasing to atmospheric pressure (day 107). From day 107 to day 115, CH_4_ production rates remained 6–8 mmol L^−1^ day^−1^ or 400–500 mg COD L^−1^ day^−1^ at a circumneutral pH. From day 115 however, at a *p*CO_2_ of 0.10 MPa and a pH 6.5, CH_4_ production rates dropped further to 2–3 mmol L^−1^ day^−1^ or 100–200 mg COD L^−1^ day^−1^ and did not recover whilst *p*CO_2_ increased to 0.25 MPa at a pH 6.3. Concomitantly, both propionate and acetate accumulated to 888 and 720 mg L^−1^ and measured *p*CO_2_ no longer corresponded to calculated *p*CO_2_ (Additional file 3: Figure S1a). By day 149, acetate concentration had decreased to 12 mg L^−1^, whilst a propionate concentration of 370 mg L^−1^ remained.

Starting period 5 (P5 day 149–157), at day 149, pressure was released to increase the pH, thereby allowing the conversion of accumulated propionate, whilst adding limited amounts of substrate, i.e. 550 mg COD L^−1^ of both acetate (516 mg L^−1^) and glucose (522 mg L^−1^). This initially led to an increase in acetate concentration from 24 (at day 149) to 912 mg L^−1^ (at day 150) and decreased afterwards to 280 mg COD L^−1^ (264 mg L^−1^; at day 154) and 21 mg COD L^−1^ (18 mg L^−1^; at day 160). Propionate gradually increased from 590 (at day 149) to 795 mg COD L^−1^ (526 mg L^−1^; at day 154). Then from day 154 onwards, propionate was removed at an estimated rate of 120 ± 10 mg COD L^−1^ day^−1^ (81 ± 7.4 mg L^−1^ day^−1^), reaching 328 mg COD L^−1^ (222 mg L^−1^) at day 158. On day 158, the headspace was flushed twice with hydrogen (an initial *p*H_2_ of 0.27 and 0.40 MPa *p*H_2_) to verify inhibition of propionate removal by *p*H_2_. The propionate concentration initially remained stable at 342 mg COD L^−1^ (229 mg L^−1^) at day 160, but subsequently decreased to 40 mg COD L^−1^ (27 mg L^−1^) at day 162 after acetate was depleted and *p*H_2_ reduced to 0.1 MPa. In period 6 (P6 day 169–192), a third autogeneration of biogas pressure started (I-17). 1100 mg COD L^−1^ (1030 mg L^−1^) glucose was provided, generating a pressure of 0.59 MPa at day 182. Propionate was again the dominant VFA, but its concentration never exceeded 300 mg COD L^−1^ (200 mg L^−1^). However, in experiment I-19, addition of 2200 mg COD glucose L^−1^ (2060 mg L^−1^) again resulted in propionate accumulation up to 800 mg COD L^−1^ (533 mg L^−1^). At a pressure of 0.84 MPa (on day 192), the experiment I was completed. In the period 192–248, it was unsuccessfully attempted to recover CH_4_ production and prevent propionate accumulation by operating at low pressure >0.20 MPa. From day 248, the focus shifted to propionate dynamics in experiment II.

### Thermodynamic feasibility

During the biological conversions the concentration of gaseous end-products and/or dissolved intermediates varied. Gibbs free energy changes were calculated (Table 2) to assess the thermodynamic limitation of end-product accumulation based on measured and calculated CO_2_ speciation (as shown in Additional file 3: Figure S1a, b). HCO_3_^−^ remained nearly constant at 150 ± 6 mmol L^−1^, whereas measured *p*CO_2_ and CO_2_(aq) varied up to 0.50 MPa and up to 135 mmol L^−1^, respectively, depending on the amount of substrate converted. Table 2 shows standard and actual Gibbs free energy change of the conversions discussed here, with the carbonic species expressed as CO_2_ and HCO_3_^−^_(aq)_.

**Table 2:** Gibbs free energy change of relevant reactions and CO_2_ speciation (based on $\Delta G_{\text{f}}^{0}$ [32])

Eq nr	Reaction equation substrates	Products	$\Delta G_{\text{r}}^{{0,{\text{a}}}}$ (kJ reaction^−1^)	$\Delta G_{\text{r}}^{\text{b}}$ (kJ reaction^−1^)	$\Delta G_{\text{r}}^{\text{c}}$ (kJ reaction^−1^)	$\Delta G_{\text{r}}^{\text{d}}$ (kJ reaction^−1^)
1*	Acetate^−^ + H_2_O	CH_4_ + HCO_3_^−^	−31.0	−25.8	−19.2	−17.5
2a**	4H_2_ + CO_2_ (g)	CH_4_ + 2H_2_O	−130.7	−12.5	−12.9	−53.5
2b**	4H_2_ + CO_2_(aq)	CH_4_ + 2H_2_O	−139.1	−12.7	−13.1	−53.7
2c**	4H_2_ + HCO_3_^−^ + H^+^	CH_4_ + 3H_2_O	−135.5	−21.0	−14.5	−55.0
3a	4H_2_ + 2CO_2_ (g)	Acetate^−^ + H^+^ + 2H_2_O	−95.0	+17.3	+3.4	−37.2
3b	4H_2_ + 2CO_2_ (aq)	Acetate^−^ + H^+^ + 2H_2_O	−111.7	+21.4	+7.5	−33.1
3c	4H_2_ + 2HCO_3_^−^ + 2H^+^	Acetate^−^ + H^+^ + 4H_2_O	−104.5	+4.7	+4.7	−35.8
4a	Propionate^−^ + 2H_2_O	Acetate^−^ + 3H_2_ + CO_2_(g)	+71.8	−19.1	−12.1	+18.3
4b	Propionate^−^ + 2H_2_O	Acetate^−^ + 3H_2_ + CO_2_(aq)	+80.1	−18.9	−11.9	+18.5
4c	Propionate^−^ + 3H_2_O	Acetate^−^ + 3H_2_ + HCO_3_^−^ + H^+^	+76.5	−10.5	−10.5	+19.9
5a	C_6_H_12_O_6_ + 2H_2_O	2 acetate^−^ + 2H^+^ + 4H_2_ + 2CO_2_(g)	−215.9	−342.0	−328.0	−287.4
5b	C_6_H_12_O_6_ + 2H_2_O	2 acetate^−^ + 2H^+^ + 4H_2_ + 2CO_2_(aq)	−199.2	−341.6	−327.6	−287.0
5c	C_6_H_12_O_6_ + 4H_2_O	2 acetate^−^ + 4H^+^ + 4H_2_ + 2HCO_3_^−^	−206.5	−324.9	−324.9	−284.3

Δ*G*_r_^0,a^ at 25 °C, pH 7 and 0.10 MPa pressure and 1 M of all aquatic species; $\Delta G_{\text{r}}^{\text{b}}$ at 30 ^°^C 0.01 M aquatic species, 0.15 M HCO_3_^−^, pH 6.2 and a *p*CO_2_ = 30 kPa and *p*H_2_ = 1 Pa; Δ*G*_r_^c^ at 30 °C 0.01 M aquatic species, 0.15 M HCO_3_^−^, pH p*K*_a_ = 6.2 and a *p*CO_2_ = 0.50 MPa and *p*H_2_ = 1 Pa; $\Delta G_{\text{r}}^{\text{d}}$ at 30 °C 0.01 M aquatic species, 0.15 M HCO_3_^−^, pH = p*K*_a_ = 6.2 and a *p*CO_2_ = 0.50 MPa and *p*H_2_ = 60 Pa; * *p*CH_4_ in Δ*G*_r_^0′a^, Δ*G*_r_^b^, Δ*G*_r_^c^ and $\Delta G_{\text{r}}^{\text{d}}$ is 0.10, 0.07, 1.00 and 2.00 MPa, respectively, ** *p*CH_4_ in Δ*G*_r_^0,a^, Δ*G*_r_^b^, Δ*G*_r_^c^ and Δ*G*_r_^d^ is 0.10, 0.07, 1.00 and 1.00 MPa, respectively

The feasibility of aceticlastic methanogenesis under prevailing conditions was calculated at CH_4_ pressure up to 2.00 MPa. In line with previous results [7, 16], accumulation from 0.07 (atmospheric) up to 2.00 MPa CH_4_ decreased the Δ*G*_r_ of aceticlastic methanogenesis from −25.8 to −17.5 kJ reaction^−1^ (Table 2; reaction 1; $\Delta G_{{_{\text{r}} }}^{{{\text{b}},{\text{d}}}}$). Likewise, hydrogenotrophic methanogenesis (Table 2; reaction 2) is also unlikely to be affected by *p*CH_4_ up to 1.00 MPa; even at 1 Pa *p*H_2_, a Δ*G*_r_ of −14.5 kJ reaction^−1^ with elevated HCO_3_^−^ was calculated ($\Delta G_{{_{\text{r}} }}^{\text{b}}$ and $\Delta G_{{_{\text{r}} }}^{\text{c}}$). At a *p*H_2_ of 60 Pa reaction 2 ($\Delta G_{{_{\text{r}} }}^{\text{d}}$) would remain very favourable. It is noteworthy that values become slightly less favourable (reaction 2a and b) when using elevated values for CO_2_ (g) or CO_2_ (aq).

For homoacetogenesis (reaction 3a), $\Delta G_{{_{\text{r}} }}^{\text{b}}$ would only be +17.3 kJ reaction^−1^ at atmospheric digester conditions (0.03 MPa *p*CO_2_ and 1 Pa *p*H_2_) whereas at 0.50 MPa *p*CO_2_ and 1 Pa *p*H_2_, Δ*G*_r_ of homoacetogenesis becomes +3.4 kJ reaction^−1^ (reaction 3a; $\Delta G_{{_{\text{r}} }}^{\text{c}}$). Although *p*CO_2_ has a positive effect on the thermodynamic favourability of homoacetogenesis, a further elevation of *p*H_2_ to 10 Pa is required for a feasible reaction (Additional file 4). The Δ*G*_r_ of propionate degradation (Table 2; reaction 4a; $\Delta G_{{_{\text{r}} }}^{{{\text{b}},{\text{c}}}}$) changes from −19.1 to −12.1 kJ mol^−1^, by elevating *p*CO_2_ from 0.03 to 0.50 MPa at an assumed *p*H_2_ of 1 Pa. This value is slightly higher than the −10.5 kJ mol^−1^ calculated using an HCO_3_^−^-based reaction equation (4c). By elevating *p*H_2_ to 60 Pa, the propionate oxidation becomes less favourable. In terms of anaerobic glucose oxidation (Table 2; reaction 5), it can be seen that although elevation of CO_2_ in any form makes the reactions less favourable ($\Delta G_{{_{\text{r}} }}^{\text{a}}$ vs $\Delta G_{{_{\text{r}} }}^{{{\text{b}},{\text{c}}}}$), a change in *p*H_2_ to 60 Pa ($\Delta G_{{_{\text{r}} }}^{\text{d}}$) largely determines the available energy.

### Population dynamics

**Fig. 3:**
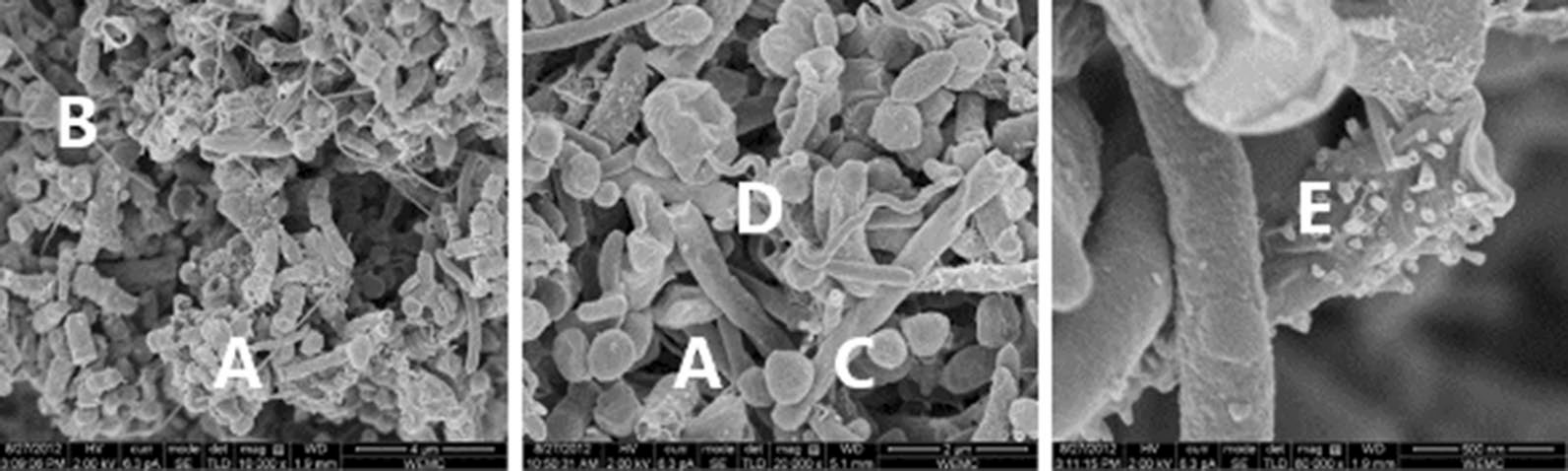
FESEM micrographs from representative reactor samples. Rod (*A*), and filamentous (*B*) shaped (*left*) and coccus (*C*), spiral-shaped (*D*) organisms (*middle*). Smooth and tubular pore (*E*) cell surfaces are magnified on the *right*

Figure 3 shows FESEM micrographs of coccus- (A), filamentous (B), rod-shaped (C) and spiral (D) microorganisms in a representative sample from the reactor biomass after completing the experiment on day 192.

The sizes varied between 0.5 and 1.0 µm diameter for the coccoid organisms, up to a width × length of 0.5 × 6 µm and 80 nm × 30 µm for the rod-shaped and filamentous organisms, respectively. The spiral organism had a width of 150 nm and a length of 8–10 µm. Cell surface appearances ranged from apparently smooth (B) to cells with tubular pores (E).

**Fig. 4:**
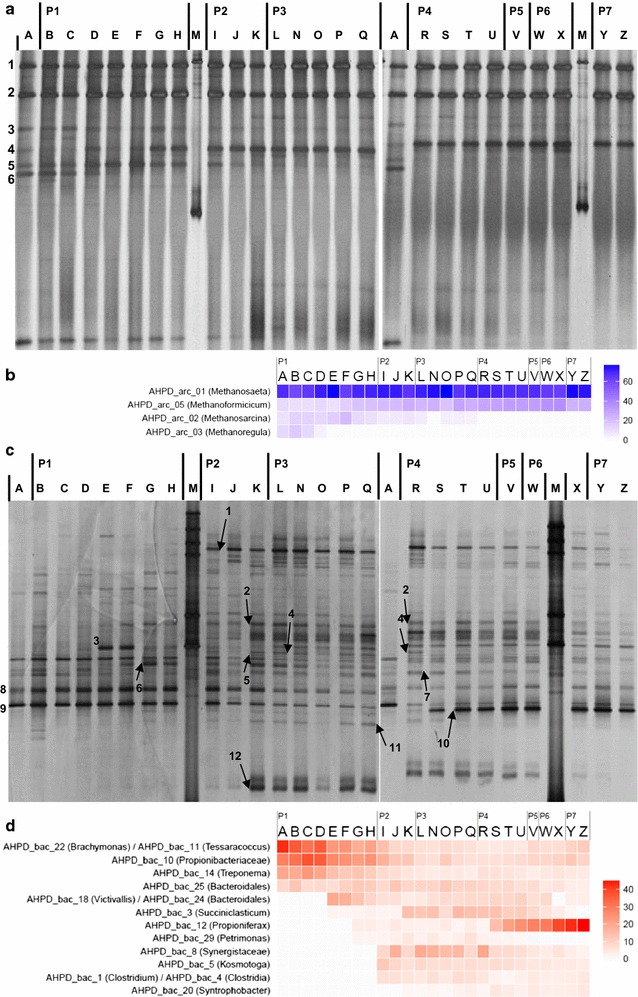
Archaeal and bacterial DGGE profiles and heat maps. Archaeal (**a**) and bacterial (**c**) DGGE profiles and heat maps of the relative intensities of major archaeal (**b**) and bacterial (**d**) DGGE bands. *Numbered bands* in **a** indicate the positions identical to the migration of clone samples closely related to (1–3) *Methanosaeta concilii*, (4) *Methanobacterium formicicum*, (5) *Methanoregula boonei* and/or *Methanosarcina acetivorans*, and (6) *Methanoregula boonei* and/or *Methanobacterium formicicum*. *Numbered bands* in **b** indicate the positions identical to the migration of clone samples closely related to (1) *Brachymonas denitrificans* and *Tessaracoccus* (2) *Propionibacteriaceae*, (3) *Treponema*, (4) *Bacteroidales*, (5) *Bacteroidales* and *Victivallis*, (6) *Succiniclasticum*, (7) *Propioniferax*, (8) *Petrimonas*, (9) *Synergistaceae*, *Brachymonas denitrificans* and *Tessaracoccus*, (10) *Kosmotoga*, (11) *Clostridium quinii* and *Clostridia*, and (12) *Syntrophobacter fumaroxidans*. Each *band* in **c** and **d** is labelled with the clone(s) with an identical migration pattern, followed in parentheses by the affiliation of the clone determined by Ribosomal Database Project classifier. *Numbers* indicate ratio (%) over the sum of band intensities of each sample (i.e., each lane in DGGE). P1–P6 and II, IV indicate operational periods and experiments described in Table 1

**Fig. 5:**
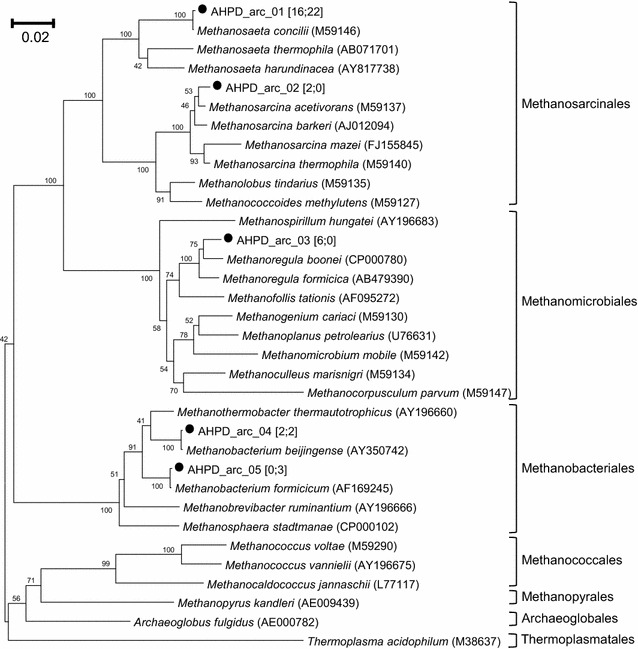
Neighbour-joining tree illustrating the phylogenetic identities of archaeal communities in the pressure bioreactor. The archaeal 16S rRNA gene fragments were obtained from clone samples. Clone counts of each OTU are given in *brackets*; the *first* and the *second numbers* indicate the counts derived from samples A and L, respectively. *Numbers* at nodes are bootstrap values derived from 100 analyses. The *scale bar* represents an amount of nucleotide sequence change of 0.02

**Fig. 6:**
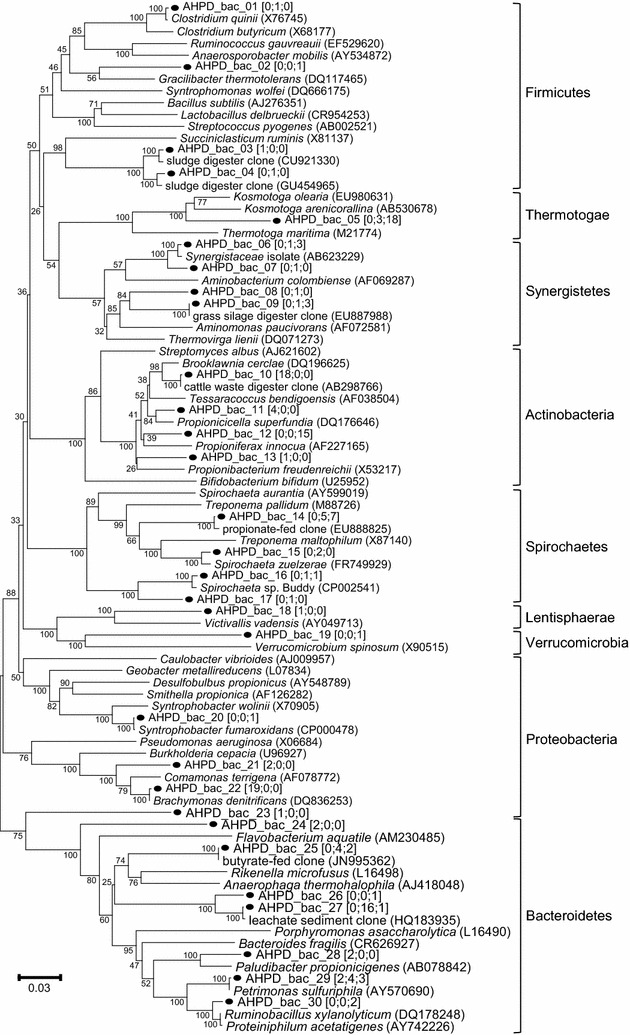
Neighbour-joining tree illustrating the phylogenetic identities of bacterial communities in the pressure bioreactor. The bacterial 16S rRNA gene fragments were obtained from clone samples. Clone counts of each OTU are given in *brackets*; *numbers* in series indicate the counts derived from samples F, L and U, respectively. *Numbers* at nodes are bootstrap values derived from 100 analyses. The *scale bar* represents an amount of nucleotide sequence change of 0.03

DGGE revealed the microbial community structure in the pressurized bioreactor (Fig. 4; Additional file 3: Figures S3, S4). Both bacteria and Archaea shifted according to temporal changes; bacteria exhibited more diverse and dynamic band patterns than Archaea. Two archaeal clone libraries were generated for sample A (the inoculum, 26 clones) and sample L (27 clones, experiment I, day 70; P3), and three bacterial clone libraries were constructed for sample F (53 clones), sample L (42 clones, experiment I, day 70; P3) and sample U (59 clones, experiment I, day 149; P4). The archaeal clones were grouped into five OTUs, whereas the bacterial clones were classified into 30 OTUs. Neighbour-joining trees showing the phylogenetic identities of the representative clones from archaeal and bacterial OTUs were constructed (Figs. 5, 6).

The five archaeal OTUs were closely (>98% 16S rRNA sequence similarity) related to *Methanosaeta concilii*, *Methanosarcina acetivorans*, *Methanoregula (Mr.) boonei*, *Methanobacterium beijingense*, and *Methanobacterium formicicum*, respectively (Fig. 5). The *Mst. concilii*-like clones represented the major population in both sample A (16/26, 62%) and L (22/27, 81%) libraries. These putative acetate-utilizing clones appeared at the same positions as bands 1–3 (Additional file 3: Figure S3), which were the most dominant in all lanes (Fig. 4). The two OTUs related to *Msr. acetivorans* and *Mr. boonei* were only present in the inoculum library. The OTU related to *Mtb. beijingense* was present in both archaeal clone libraries. The DGGE bands associated with these clones became less prominent with time (Fig. 4), indicating that the relative abundance of these species decreased with time. The *Mtb. formicicum*-like clones, in contrast, were only detected in sample L (3/27, 11%) but not in the inoculum sample (A). The corresponding DGGE band faded and became prominent from sample F onwards (Fig. 4), implying that the *Mtb. formicicum*-related archaeon was one of the dominant hydrogen-utilizing methanogens during the pressurized operation.

The 30 bacterial OTUs were affiliated to nine phyla: *Firmicutes*, *Thermotogae*, *Synergistetes*, *Actinobacteria*, *Spirochaetes*, *Lentisphaerae*, *Verrucomicrobia*, *Proteobacteria*, and *Bacteroidetes* (Fig. 6). Amongst these, 15 OTUs matched to 12 bands with identical mobility on DGGE (Fig. 4; Additional file 3: Figure S4). The top row in the bacterial heat map (Fig. 4; Additional file 3: Figure S4, band 1) was the most dominant in the inoculum and the acetate-fed lanes, but gradually lost its intensity afterwards. This band is linked to a group of clones closely related to *Brachymonas denitrificans*, a denitrifying bacterium [34], or to *Tessaracoccus* spp., a polyphosphate-accumulating bacterium [35]. These OTUs respectively accounted for 36% (19/53) or 8% (4/53) of the sample F library but none of the other two libraries, supporting the observation from the band patterns.

Band 2, identified as a *Propionibacteriaceae*-like organism (clone AHPD_bac_10), was present from the reactor start up, but decreased its intensity from period 3 onwards. Band 3 was linked to a *Treponema*-like OTU (clone 14); this genus consists of multiple species including the homoacetogenic *T. primitia* [36]. It peaked during P1 and remained relatively stable throughout the later periods.

Three other bands (4, 5, and 11), whose intensities increased and then decreased with time, showed the same migration on DGGE to clones closely related to *Bacteroidales* (clones 24 and 25), *Victivallis* (clone 18), *Clostridium quinii* (clone 1), and/or *Clostridia* (clone 4). Clones 24 (*Bacteroidales*) and 18 (*Victivallis*), both appeared at the same position in the DGGE and thus no distinction could be made.

Band 6 (clone 3), related to *Succiniclasticum*, appeared at the end of P2, but decreased in intensity from P4 onwards (Fig. 4; Additional file 3: Figure S4). The propionate-producing *Propioniferax*-like species (clone 12 and band 7) was only retrieved in the clone library of sample U with 25% of the total counts (15 of 59 clones). The fact, together with the high intensity of band 7 shown from sample S (day 112) onwards, seems to indicate that the observed propionate accumulation and the dominance of this *Propioniferax*-like species in the bacterial community are interrelated.

It is also noteworthy that band 8 (clone 29), which was identified as a *Petrimonas*-related clone, appeared during the period of pressure operation (P2), but showed diminished intensity after pressure decreased to below 1.0 MPa.

Clone AHPD_bac_8 (band 9), which was deeply related within Synergistaceae, appeared at the time when the substrate was changed from acetate to glucose (Fig. 4; Table 1) and remained visible throughout the pressure operation. Band intensity decreased in P4 when propionate accumulation started.

A *Kosmotoga*-affiliated clone (AHPD_bac_5) constituted 7% (3/42) and 31% (18/59) of the clones of sample L (experiment I, day 70; P3) and sample U (experiment I, day 149; P4) libraries, respectively. Considered together with the appearance of the related band (band 10) from period 2, this *Kosmotoga*-related phylotype seems to have developed as one of the dominant bacterial species (Fig. 4).

Clone AHPD_bac_20 showed 99% 16S rRNA sequence identity to *Syntrophobacter fumaroxidans*, a propionate oxidizer, and only one clone was retrieved from sample U. Accordingly, the intensity of the related DGGE band (band 12) appeared during period 2 and was relatively weak throughout the experiment. This result implies that the relative abundance of propionate oxidizers was low in the AHPD reactor.

### Propionate kinetics

In experiment II (Table 1), it was hypothesized that a temporary increase in *p*H_2_ would stimulate interspecies hydrogen transfer by thermodynamically favouring the syntrophic partners of the propionate-oxidizing organisms [37]. First, propionate accumulation was achieved by adding glucose (2200 mg COD L^−1^; 2060 mg L^−1^). Then, the hydrogen partial pressure in the reactor was increased in two subsequent stages to 0.27 and 0.40 MPa by manually adding hydrogen from a pressurized hydrogen bottle (Additional file 3: Figure S2a). Acetate accumulated (Additional file 3: Figure S2b), whilst *p*CO_2_ decreased and *p*CH_4_ increased. Partial consumption of H_2_ was also observed, but the system remained pressurized with a *p*H_2_ of over 10 kPa, whilst propionate oxidation commenced (Additional file 3: Figure S2b). CO_2_ and CH_4_ (0.15 MPa) production indicated methanogenic activity. Results presented in Additional file 3: Figure S2b indicated that despite the earlier observed propionate accumulation, the presence of an active homoacetogenic and/or hydrogenotrophic population was confirmed. After flushing the remaining H_2_ with N_2_, both acetate and propionate were gradually removed revealing the presence of syntrophic and acetotrophic communities.

**Fig. 7:**
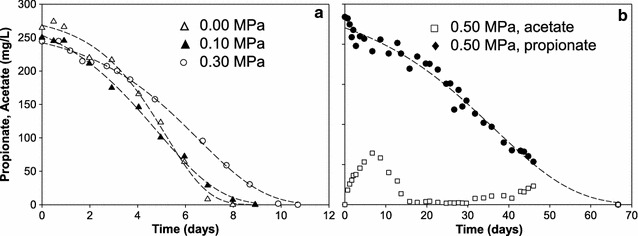
Results of the propionate degradation experiments (experiment III) under different *p*CO_2_ conditions. **a** Propionate degradation profiles under different *p*CO_2_ conditions. **b** Both acetate and propionate profiles of 0.50 MPa trial are shown for representation. *Dashed lines* represent curve fittings using modified Gompertz model

From these results, we suspected a possible inhibitory role of CO_2_ on propionate oxidation. In experiment 3, we tested this hypothesis of *p*CO_2_ induced inhibition of propionate conversion under 0.00, 0.10, 0.30 and 0.50 MPa *p*CO_2_ (experiment III; Table 3; Fig. 7a, b). The raw data for Fig. 7 can be found in Additional file 5.

**Table 3:** Kinetic parameters derived from the propionate degradation experiment

Parameter	*p*CO_2_	0	1	3	5
	pH	7.8	7.1	6.3	6.1
*A* (mg L^−1^)		283	283	266	258
*λ* (days)		2.8	3.4	3.8	16.8
*r*_smax_ (mg L^−1^ day^−1^)		72.8	58.5	35.5	4.8
Reactor *r*_smax_ (mg COD L^−1^ day^−1^)^b^		546	441.5	268	36
Specific *r*_smax_ (mg g^−1^ VS_added_ day^−1^)		30.3	24.4	16.5	2.2
Relative *µ*_max_ (%)^a^		100	80.5	54.5	7.3

All *p* values are <10^−4^

*A* = initial substrate concentration in mg L^−1^; *λ* = lag phase in days

^a^Calculated by assuming constant yield coefficient in different experiments

^b^Given five times dilution of reactor sludge concentration

Kinetic parameters for propionate conversion were derived from the modified Gompertz model [19, 38] and are listed in Table 3. The lag period (*λ*) increased with higher *p*CO_2_ whilst the maximum conversion rate (*r*_smax_) decreased; thus, providing clear evidence that an elevated *p*CO_2_ had detrimental effects on anaerobic propionate catabolism. The 0.50 MPa experiment showed significant (*p* < 10^−4^) reduction (93%) of the maximum conversion rate compared to the 0.00 MPa *p*CO_2_ experiment. Besides propionate, acetate was the only VFA detected in this experiment. Acetate profiles of the 0.50 MPa experiment are represented in Fig. 7b. Acetate accumulated to 68 mg COD L^−1^ (64 mg L^−1^) during the lag period, decreased afterwards and maintained at low levels during the entire active propionate conversion period.

To better understand the mechanism behind the decreased propionate conversion, an additional experiment was performed in which pH was reduced by means of HCl addition and by *p*CO_2_ to 6.3. From Additional file 3: Figure S5, it can be observed that in the HCl -controlled experiment 1812 mg COD L^−1^ (1200 mg L^−1^) propionate was degraded in all duplicate experiments within 6 days. Yet, at elevated *p*CO_2_ experiment (IV-4) 906 mg COD L^−1^ (600 mg L^−1^) propionate was left after 7 days. It is therefore unlikely that the decreased propionate conversion can be explained by decreased pH alone (Additional file 3: Figure S5).

## Discussion

### Shifts in population dynamics by long-term exposure to AHPD conditions

It was found that over time and concomitantly with longer exposure to elevated *p*CO_2_ a shift occurred from acetate to propionate as the main accumulating intermediate from glucose degradation. *Kosmotoga*-affiliated clone 5 constituted 7% (3/42) and 31% (18/59) of the clone counts of the L (experiment I, day 70; P3) and U (experiment I, day 149; P4) libraries, respectively. Considered together with the first visualization of band 10 during period 2 (Fig. 4; Additional file 3: Figure S4), this *Kosmotoga*-related organism developed as one of the dominant bacterial species under the pressure conditions of period 3, where acetate was the main intermediate. However, its relative band intensity decreased from sample R onwards, when higher transient propionate was observed. The only mesophilic member of this genus, *K. olearia*, was isolated from oil production fluid produced at an oil platform in the North Sea, which is characterized by an outer sheath-like structure or ‘toga’ and as an acetate and hydrogen producer [39]. Likewise, the *Synergistaceae*-related band (clone 8; Fig. 4) showed relatively high intensity during the initial pressure operation. It is noteworthy that these organisms are also characterized by a diderm atypical cell envelope [40]. *Clostridium quinii* (the closest relative of clone 1) and *Petrimonas sulfuriphila* (the only characterized species of this genus; clone 29) are both sugar-utilizing microorganisms producing acetate and hydrogen as common products [41, 42]. The genus *Victivallis* (clone 18) includes only one isolated species, *V. vadensis*, which converts glucose to acetate and H_2_ in a syntrophic co-culture with hydrogen-utilizing methanogens [43].

Besides acetate producers, also propionate producers were found in the clone libraries. *Succiniclasticum* (clone 3) includes a sole member, *S. ruminis*, which is known to convert succinate to propionate [44]. *Propionibacteriaceae* (clone 10) are well recognized for its sub-lineage *Propionibacteria* spp. which produce propionate via the Wood-Werkman cycle in anaerobic digesters [45]. The AHPD conditions in this study seemed to be unfavourable for these organisms, because the corresponding band 2 (Fig. 4; Additional file 3: Figure S4) faded from sample L onwards, with no clones found in samples L (experiment I, day 70; P3) and U (experiment I, day 149; P4). In contrast, conditions favoured the growth of a propionate producing *Propioniferax*-like organism (clone bac_12; Fig. 4; Additional file 3: Figure S4, band 7). The abundance of these organisms, as evidenced by the clone counts and band prominence from sample *R*, suggests that the presence of this organism was strongly related to the accumulation of propionate under the tested conditions. Strikingly, the increase in band intensity (Fig. 4) of the *Propioniferax*-like organism was accompanied by the decrease in band intensity of the *Kosmotoga*-like organism (clone bac_5: Fig. 4). Apparently, both organisms compete for glucose under the given conditions.

With regard to acetate conversion, it can be observed that *Msr. acetivorans*-like archaeon became prominent under the initial acetate feeding (Fig. 3a). However, after switching to glucose digestion it progressively disappeared until the end of period 3 when the highest pressures of this study were obtained. The *Mst. concilii*-like species appeared to be the most abundant archaeon throughout the further operation.

The kinetic competition for acetate utilization between *Methanosaeta* and *Methanosarcina* spp. is well documented [46, 47]. In an acetate fed-batch incubation harbouring the two genera, the r-strategist *Methanosarcina* typically outcompetes the K-strategist *Methanosaeta* at high acetate concentrations (>120 mg COD L^−1^; 114 mg L^−1^). From period 2 onwards, intermediate acetate concentrations maintained below 120 mg COD L^−1^ (114 mg L^−1^), except within 1–3 days after feeding glucose. From the end of period 3 (sample L), intermediate acetate concentrations also remained low. The filamentous structure [48] of *Mst. concilii* could have mitigated the dominance of this species at elevated *p*CO_2_ due to its higher surface-to-volume ratio, which could strengthen the influence of *p*CO_2_ and carbonic species. Thus, the *Kosmotoga*-like organism (clone bac_5), with clone counts increasing from 7 to 31% in samples L (experiment I, day 70; P3) and U (experiment I, day 149; P4), respectively, appears to have been involved in the well-balanced acetate formation and consumption with *Mst. concilii*.

Like acetate, propionate is an important intermediate in the anaerobic food chain through which 6–35% of the electron equivalents are channelled under atmospheric conditions by enriched methanogenic cultures [49]. Elevated levels of propionate are often regarded as a sign of digester instability due in part to its toxicity [50] and especially to its critical thermodynamics for anaerobic conversion [37, 51, 52]. Although propionate oxidation seemed to occur readily up to a pressure of 2.00 MPa and pH 6.1 (P3) with concentrations below 400 mg COD L^−1^ (267 mg L^−1^), detrimental accumulation of propionate, coinciding with partial inhibition of methanogenesis, occurred in P4 and P6 experiments. The *Syntrophobacter fumaroxidans*-like clone (clone 20; 99% sequence identity) was the solely identified propionate utilizer in this experiment, and was counted only once in sample U and its related band 12 became weak in intensity. This implies that the propionate oxidation under AHPD conditions was carried out, at least partially, by a propionate oxidizer, which is commonly observed under non-pressurized conditions. Nevertheless, it should be realized that these culture-independent methods, DGGE and clone library analyses, do not support direct evidence on the population size or activity and are subject to PCR bias [53]. Employment of additional techniques, such as fluorescence in situ hybridization, radiography, polyomics approaches, or culture-based methods, would provide multi-dimensional insights to further elucidate population dynamics. Another possibility is that other organisms were responsible for propionate oxidation. Clone AHPD_bac_14, for example, could have been involved in propionate oxidation, as it showed highest similarity (99%) to a clone (EU888825) retrieved from a propionate-fed anaerobic reactor [54].

Data from reactor operation in P4, P5 and P6 showed stable or increasing propionate concentrations directly after glucose feeding; besides increased propionate production, this could indicate decreased propionate consumption. However, propionate conversion rates of up to at least 250–300 mg COD L^−1^ day^−1^ (165–198 mg L^−1^ day^−1^) were also observed in P4 and P5 about 4 days after glucose was fed. It was therefore confirmed that an active propionate-degrading community was still present, although it could not prevent propionate accumulation. The microbial diversity analysis confirmed the continued presence of a stable hydrogen-consuming population. Next to the hydrogenotrophic methanogens, *Mtb. formicicum* and *Mtb. beijingense*, the presence of a *Treponema*-like bacterium (clone 14) was shown. This genus harbours many species including the hydrogen-consuming acetogenic *T. primitia* [36]. H_2_ was never detected above the instrument’s detection limit of 60 Pa in the gas phase, but calculations (Table 2) show that propionate oxidation is strongly inhibited below 60 Pa. Nevertheless, propionate was oxidized in experiment II at elevated *p*H_2_ of 0.27 and 0.40 MPa (Additional file 3: Figure S2). Under even higher *p*H_2_, this is only feasible with an active syntrophic community keeping *p*H_2_ in the proximity of propionate-oxidizing organisms extremely low and is comparable to the thermophilic propionate conversion kinetics observed elsewhere [51]. This allows us to exclude the possibility that the mixing profile had disturbed the granules structure and still provided the required proximity for interspecies hydrogen transfer. It cannot be excluded, however, that temporary increases in *p*H_2_ as small as 1 Pa resulting from rapid glucose degradation could have reduced the thermodynamic favourability of syntrophic propionate oxidation, transiently resulting in a lower propionate oxidation rate. This is a critical aspect of high-pressure digestion of sugars and therefore requires further investigation. It might also be of interest for stimulating undefined mixed-culture propionate fermentations within the carboxylate platform [55].

### Potential mechanisms for the observed propionate conversion inhibition by *p*CO_2_

The inoculum for experiment III was taken from the 8-L reactor at a *p*CO_2_ exceeding 0.30 MPa, implying that the consortia might have adapted to higher CO_2_ levels. Nevertheless, experiment III showed that with increasing *p*CO_2_ (Table 3; 0.1–0.5 MPa) the specific propionate oxidation rate decreased linearly from 45.8 to 3.3 mg COD (30.3–2.2 mg) g VS^−1^ day^−1^. Both values are within the 1.4–755 mg COD (0.74–503.2 mg) g^−1^ VSS day^−1^ range for specific propionate conversion described in previous studies [56–60]. The very low rates of 3.3 mg COD (2.2 mg) g VS^−1^ day^−1^ at 0.50 MPa *p*CO_2_ are similar only to rates found for extremely high solid digestion (65 or 75% moisture content) [57]. In experiment I-14 propionate was oxidized (after all glucose was consumed) at an estimated rate of ~60 mg COD L^−1^ day^−1^ (40 mg L^−1^ day^−1^) at a *p*CO_2_ of 0.25 MPa and estimated CO_2_ (aq) of 110 mmol L^−1^. In experiments I-15 and 16, when *p*CO_2_ was below 0.1 MPa, propionate degraded at an estimated rate of 120 mg COD L^−1^ day^−1^ (81 mg L^−1^ day^−1^). Although this suggests a reversible inhibition caused by CO_2_ accumulation, Additional file 3: Figure S5 clearly shows that there is also a pH-related effect. It has been demonstrated that a pH drop from 6.8 to 6.2 inhibited propionate conversion [61]. It is remarkable that the HCl-induced pH drop in experiment IV-3 did not inhibit the conversions and therefore results suggest that the observed reversible inhibition is related to the pH-based speciation of CO_2_.

On one hand, autogenerated *p*CO_2_ (of 0.03 up to 0.50 MPa) is unfavourable for the thermodynamic feasibility of propionate oxidation by shifting $\Delta G_{\text{r}}^{{^{\text{b}} }}$ from −19.1 to $\Delta G_{\text{r}}^{{^{\text{c}} }}$ −12.1 kJ mol^−1^ (Table 2 reaction 4a and Additional file 3: Figure S1a). On the other hand, it also provides excess electron acceptor for CO_2_ reducers, thereby decreasing the $\Delta G_{\text{r}}^{{^{\text{b}} }}$ of the hydrogenotrophic and homoacetogenic pathways at 1 Pa *p*H_2_ from −12.5 and +17.3 to $\Delta G_{\text{r}}^{{^{\text{c}} }}$ −12.9 and +3.4 kJ reaction^−1^, respectively (Table 2, reaction 2c and 3a). This slightly improves the conditions for interspecies hydrogen transfer and in turn enhances propionate conversion. An energetic minimum of −20 kJ mol^−1^, corresponding to 1/3 ATP, is generally needed to sustain life [32], but the continuous production of CH_4_ up to 9.00 MPa [7] would thermodynamically not have been possible with a Δ*G*_r_ of −13.1 kJ mol^−1^. Changes in free energy could theoretically affect kinetics and thereby cause the observed phenomena [62, 63], but we consider it unlikely that these minor changes with a positive feedback-loop could have caused a >90% decrease in observed propionate oxidation rates in a linear manner. In fact, many sources in literature [13, 14, 30, 64–66] show clear evidence that CO_2_ results in a pH effect, rather than only being a substrate, intermediate and end-product in free energy calculations. Even stronger effects of carbonic acid than could be explained from [H^+^] alone were reported [65]. The data presented in Additional file 3: Figure S5 support this finding also for this specific pressure cultivated sludge. Titration of the pH to 6.3 by HCl resulted in a limited inhibition compared to reaching this pH by *p*CO_2_. This gives rise to speculation on combined pH–*p*CO_2_ effects, which opens new perspectives to produce VFA for the carboxylate platform at relatively higher pH as CH_4_ production was inhibited up till pH 6.5. Potentially, CO_2_-induced inhibition could also be of interest to enrich the biocathode communities in microbial electrosynthesis (MES), without focusing on expensive pure cultures or lengthy enrichment procedures, as it was demonstrated that higher coulombic efficiencies can be reached using enriched or pure cultures instead of conventional mixed cultures [67, 68].

At increasing *p*CO_2_ and decreasing pH, CO_2_ possibly binds to the amine groups of proteins forming carbamino-proteins, potentially inhibiting an enzyme. More severe effects would be expected at pH values close to or lower than the p*K*_a_ (~5.5) of some known carbamino-proteins [69]. The formation of carbamino-proteins was reported to cause reversible sol–gel interactions in the cytoplasm of single-cell organisms, for example the filamentous algae *Nitella clavata* [64]. However, rapid or excessive increase in *p*CO_2_ caused irreversible damage to the cell structure [14]. It has been concluded that Gram-positive bacteria are more resistant towards elevated *p*CO_2_ than Gram-negative bacteria [13]. A thick peptidoglycan cell wall offers a better barrier to prevent CO_2_ diffusion into the protoplasma than an open lipopolysaccharide membrane combined with a thin peptidoglycan inner membrane. Interestingly, the Gram-positive *Propioniferax* was renamed from *Propionibacterium innocuum* to *Propioniferax innocua*, because of the exceptional cell wall structure [70]. Likewise, the *Kosmotoga*-like organism sets itself aside from other putative acetate producers by being closely related to the only mesophilic member of the *Thermotogales*, characterized by an additional protective outer envelope, the so-called Toga [39]. Although being different in composition, the thicker cell wall of Archaea probably offers more protection towards pressure as well. It seems that the microorganisms that grew in the AHPD reactor have structural adaptations to survive high pressure and high CO_2_ conditions. More fundamental research is needed to further investigate the selectivity of *p*CO_2_ toxicity.

## Conclusions

This study showed that the methanogens *Mst*. *concilii* and *Mtb. formicicum* were piezo-tolerant and were the dominant archaeal species during the autogeneration of 2.00 MPa of biogas (with 80% CH_4_) from glucose. The bacterial diversity analysis indicated that a *Propioniferax*-like organism, a *Kosmotoga*-like organism, and a *Treponema*-like organism became the dominant bacterial species under AHPD conditions, but the organisms responsible for propionate conversion could not be identified. The closest neighbours to the identified Archaea and Bacteria include piezo-tolerant and piezophilic organisms sourced from deep-sea, gas, oil and coal-bed reservoirs. AHPD experiments therefore provide an interesting tool to unravel the origin and population dynamics of biogenic natural gas.

After prolonged operation, propionate conversion became rate-limiting for methane production. It was confirmed that not *p*H_2_ but *p*CO_2_ caused the accumulation of propionate in the AHPD system. From literature three potential mechanisms were identified: (1) thermodynamic favourability, (2) pH and (3) reversible enzyme inhibition by formation of carbamino-proteins under elevated *p*CO_2_.

Thermodynamic calculations showed that this inhibition could not be explained by the relatively small changes in thermodynamic favourability. Based on our experimental results also a simple pH effect proved unlikely. Since the elevated *p*CO_2_ resulted in a selective inhibition of propionate conversion, it is highly interesting from a carboxylate production perspective to study reversible enzyme inhibition under elevated *p*CO_2_.

## Additional files

**Additional file 1: Table S1.** Overview of used values for enthalpy of formation (Δ*H*_f_^o^) and free energy of formation (Δ*G*_f_^o^) [31].

**Additional file 2.** Raw reactor data for experiment I.

**Additional file 3: Figure S1.** Results of fed-batch reactor operation. **Figure S2.** Results of fed-batch reactor operation during experiment II. **Figure S3.** Archaeal DGGE profiles of the 16S rRNA gene fragments. **Figure S4.** Bacterial DGGE profiles of the 16S rRNA gene fragments. **Figure S5.** Results of propionate degradation during experiment IV.

**Additional file 4.** Thermodynamic calculation tool.

**Additional file 5.** Raw reactor data for propionate kinetic experiment.

## Abbreviations

AHPD: autogenerative high-pressure digestion
ANC: acid-neutralizing capacity
COD: chemical oxygen demand
DGGE: denaturing gradient gel electrophoresis
EDX: energy dispersive X-ray
FeSEM: field emission scanning electron microscope
GC: gas chromatograph
HPLC: high-performance liquid chromatography
OTU: operational taxonomic unit
rcf: rotational centrifugal force
TAE: tris base, acetic acid and EDTA
TLD: through lens detection
TS: total solids
TSS: total suspended solids
VFA: volatile fatty acid
VS: volatile solids
VSS: volatile suspended solids
