# Supplementary material for: Piezo-tolerant natural gas-producing microbes under accumulating pCO2
Source: Biotechnol Biofuels. 2016 Nov 4;9:236. doi: 10.1186/s13068-016-0634-7 (PMC5097443; doi:10.1186/s13068-016-0634-7)
Supplement: Supplementary file 1 — Additional file 1: Table S1. Overview of used values for enthalpy of formation (ΔH fo) and free energy of formation (ΔG fo) [31]. [file 13068_2016_634_MOESM1_ESM.docx]

Table S1

Overview of used values for Enthalpy of formation (ΔH_f_^o^) and Free energy of formation (ΔG_f_^o^) [31].

|  | ΔG_f_^o^ (kJ mol^-1^) | ΔH_f_^o^ (kJ mol^-1^) |
| --- | --- | --- |
| C_6_H_12_O_6_ | -917 | -1264 |
| CH_3_COO^-^ | -369,41 | -486 |
| CH_3_CH_2_COO^-^ | -361,08 | -510 |
| HCO_3_^-^ | -586,85 | -692 |
| CO_2_ (g) | -394,359 | -394 |
| CO_2_ (aq) | -386,02 | -414 |
| H_2_ | 0 | 0 |
| H_2_O | -237,178 | -286 |
| CH_4_ | -50,75 | -74,8 |
| H^+^(pH=0) | 0 | 0 |
