# Supplementary material for: Piezo-tolerant natural gas-producing microbes under accumulating pCO2
Source: Biotechnol Biofuels. 2016 Nov 4;9:236. doi: 10.1186/s13068-016-0634-7 (PMC5097443; doi:10.1186/s13068-016-0634-7)
Supplement: Supplementary file 3 — Additional file 3: Figure S1. Results of fed-batch reactor operation. Figure S2. Results of fed-batch reactor operation during experiment II. Figure S3. Archaeal DGGE profiles of the 16S rRNA gene fragments. Figure S4. Bacterial DGGE profiles of the 16S rRNA gene fragments. Figure S5. Results of propionate degradation during experiment IV. [file 13068_2016_634_MOESM3_ESM.docx]

Additional file 2


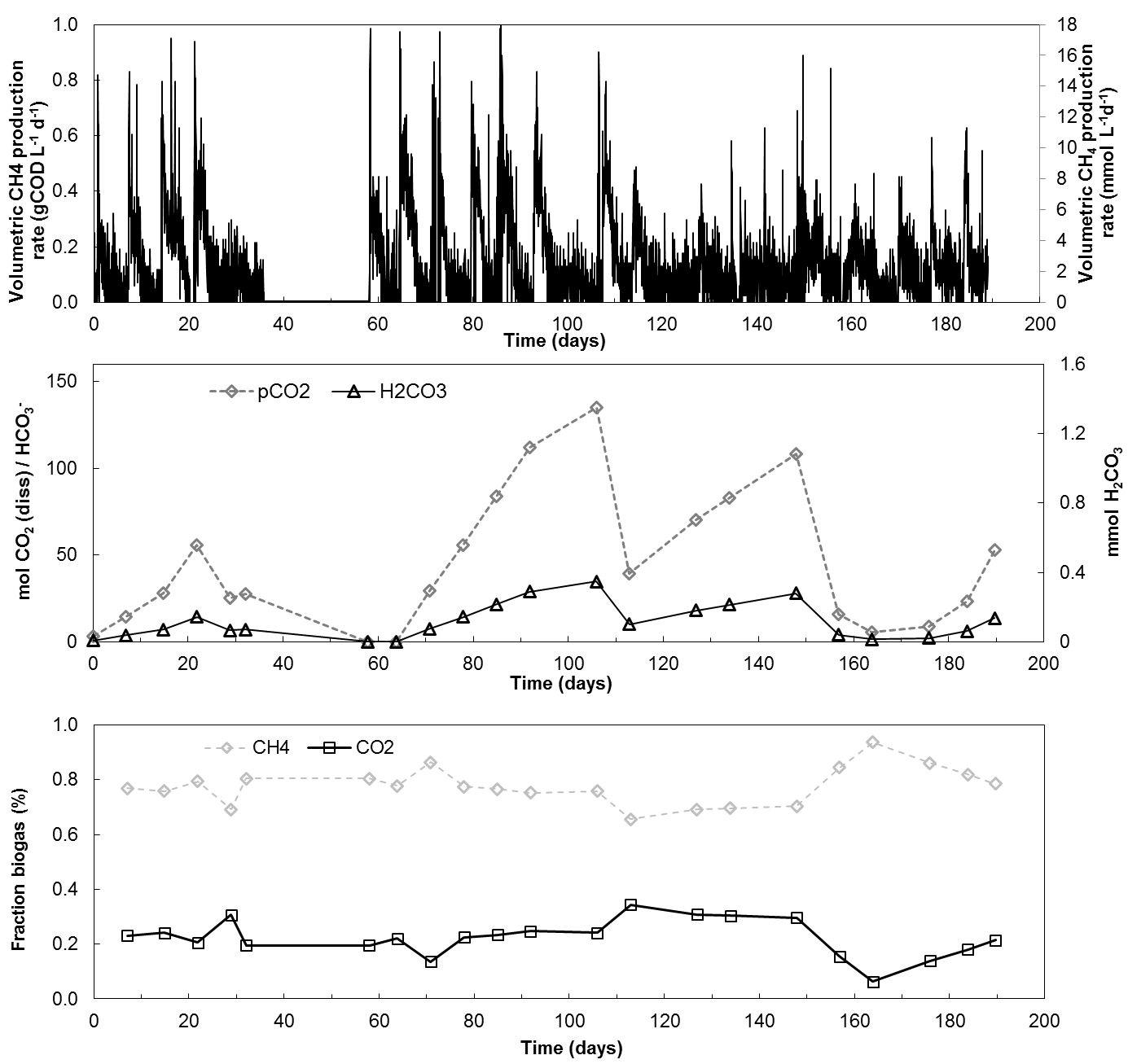


Figure S1. Results of fed-batch reactor operation.

(A) volumetric CH_4_ –production rates based on average CH_4_-content of biogas (78%) (-), calculated pCO_2_ (□) and measured pCO_2_ (◦) and (B) calculated dissolved CO_2_ (◊) and H_2_CO_3_ (∆) profiles and (C) fraction CH_4_ (◊) and CO_2_ (□) in biogas and pH (Δ) over time.


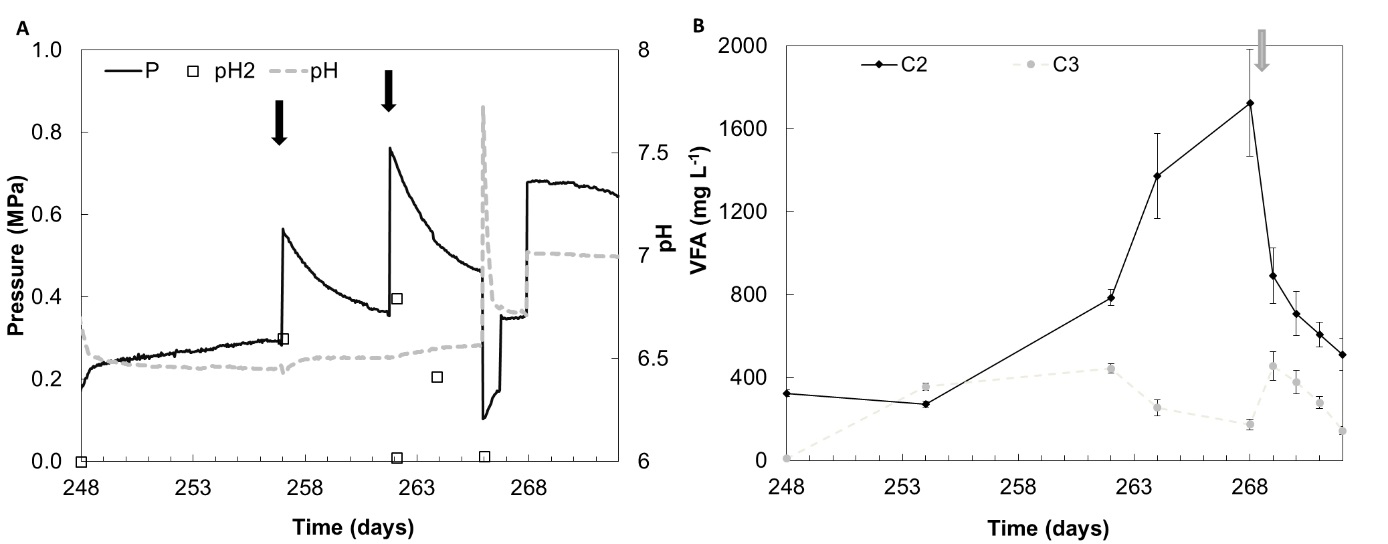


Figure S2. Results of fed-batch reactor operation during experiment II.

(A) Pressure and pH profile for experiments II-1, II-2 and II-3 with hydrogen additions (black arrows) of 0.27 and 0.40 MPa pH_2_ (open squares) and (B) related propionate (grey) and acetate (black) concentrations with nitrogen flushing (grey arrow).


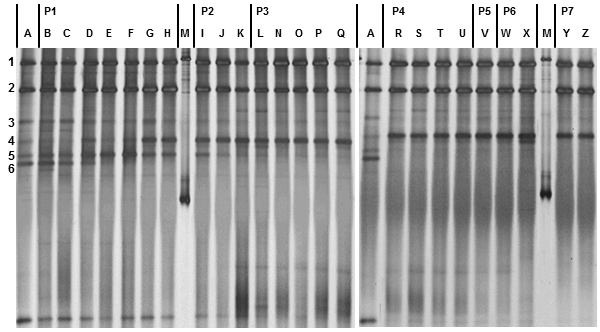


Figure S3. Archaeal DGGE profiles of the 16S rRNA gene fragments.

Numbered bands indicate the positions identical to the migration of clone samples closely related to (1–3) *Methanosaeta concilii*, (4) *Methanobacterium formicicum*, (5) *Methanoregula boonei* and/or *Methanosarcina acetivorans*, and (6) *Methanoregula boonei* and/or *Methanobacterium formicicum*.


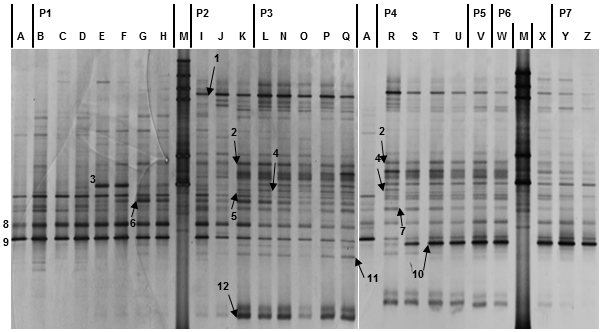


Figure S4. Bacterial DGGE profiles of the 16S rRNA gene fragments.

Numbered bands indicate the positions identical to the migration of clone samples closely related to (1) *Brachymonas denitrificans* and *Tessaracoccus* (2) *Propionibacteriaceae*, (3) *Treponema*, (4) *Bacteroidales*, (5) *Bacteroidales* and *Victivallis*, (6) *Succiniclasticum*.(7) *Propioniferax*, (8) *Petrimonas*, (9) *Synergistaceae*,*Brachymonas denitrificans* and *Tessaracoccus*, (10) *Kosmotoga*,(11) *Clostridium quinii* and *Clostridia*, and (12) *Syntrophobacter fumaroxidans*,


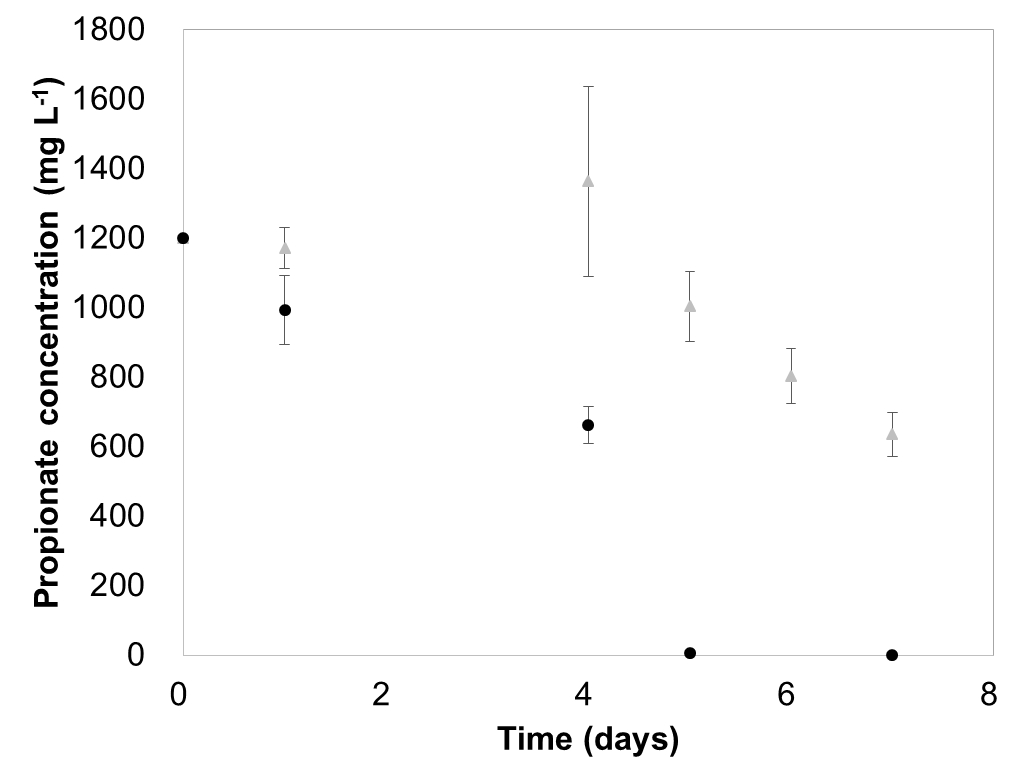


Figure S5. Results of propionate degradation during experiment IV.

Degradation of propionate in time at 0.1 MPa pN_2_ and pH 6.3 (black) and 0.6 MPa pCO_2_ and pH 6.2 (grey). *T=0 concerns the dosed propionate concentration, not measured.
